# Mental tasks induce common modulations of oscillations in cortex and spinal cord

**DOI:** 10.1186/s12984-026-02041-3

**Published:** 2026-07-11

**Authors:** Patrick Ofner, Dario Farina, Carsten Mehring

**Affiliations:** 1 Bernstein Center Freiburg, University of Freiburg, Freiburg im Breisgau, 79104 Germany; 2 Faculty of Biology, University of Freiburg, Freiburg im Breisgau, 79104 Germany; 3 Department of Bioengineering, Imperial College London, London, W12 0BZ UK

**Keywords:** Spinal motor neurons, Motor units, Beta oscillations, Mental tasks, EEG, HD-EMG, Movement augmentation, Neural interface

## Abstract

**Background:**

Spike trains from spinal motor neurons contain low-frequency components that modulate muscle force, and higher-frequency components (above 10 Hz) that do not. The functional role of these higher-frequency components in motor control is still debated. We investigated whether mental tasks that modulate the power of cortical oscillations produce corresponding modulations in spinal motor neuron activity above 10 Hz without affecting force output. Such coupling would indicate that some higher-frequency components are not merely arising as a byproduct of force generation nor indirectly contributing to motor control, but simply reflect cortical oscillations propagating to spinal motor neurons. If voluntary power modulations of these higher-frequency oscillations do not affect force output, they could potentially serve as control signals for neural interface applications such as movement augmentation or motor neuroprostheses.

**Methods:**

We recruited 15 human participants and recorded high-density electromyography signals (HD-EMG) from the tibialis anterior muscle, as well as electroencephalography (EEG) signals. The cumulative spike train (CST) was computed from the activity of spinal motor neurons decoded from HD-EMG signals. The participants performed sustained dorsiflexion concurrent with foot motor imagery, hand motor imagery, mental arithmetic, or no specific mental task. We analysed the bandpower correlation between EEG and CST signals as well as evaluated the task discriminability of CST bandpower signals with a linear classifier.

**Results:**

At the intra-muscular coherence peak, we found statistically significant power correlations between CST and EEG in two separate analyses: first, when correlating across individual trials regardless of the mental task, and second, when correlating across the four mental tasks (Kendall’s $\tau$ coefficient $\tau _{trial} = 0.08 \pm 0.10$, $\tau _{task} = 0.33 \pm 0.19,$ respectively; mean ± std. dev.). To evaluate the potential of the CST as a control signal, we classified the mental tasks based on CST bandpower and obtained classification accuracies slightly but significantly above chance level ($30\% \pm 5\%$; chance level = 25%).

**Conclusion:**

These results show that mental tasks can simultaneously modulate the power of cortical and spinal oscillations. This supports the notion that cortical oscillations not contributing to ongoing force control can propagate to the spinal level. We further demonstrate that mental tasks can be classified from CST bandpower, but classification performance is limited by the low signal-to-noise ratio.

## Introduction

**Fig. 1:**
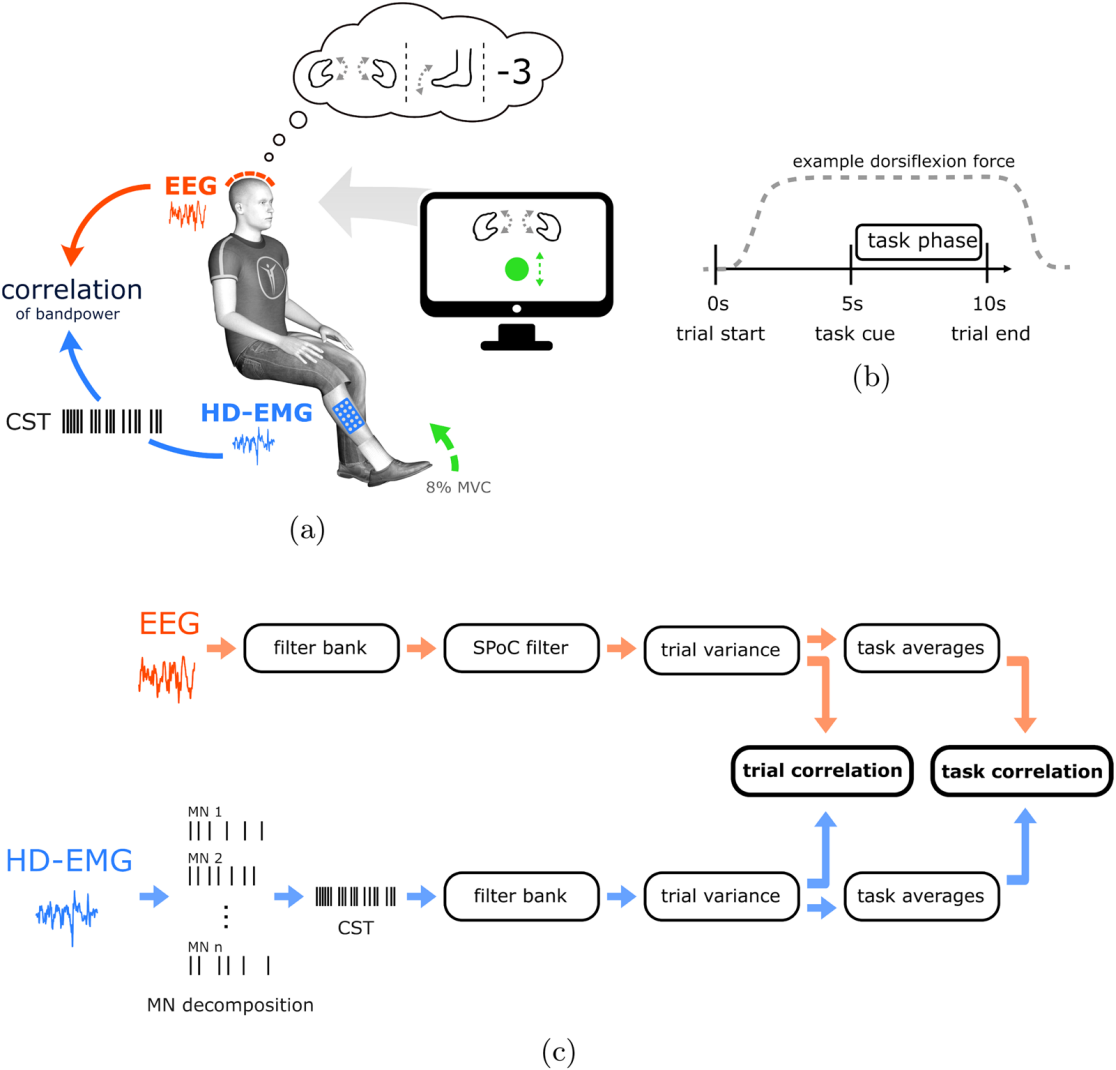
**a** Experiment setup. HD-EMG and EEG were recorded while participants performed an isometric dorsiflexion at 8% MVC together with a mental task (hand MI, foot MI, mental arithmetic, or no task). The computer screen displayed task instructions and provided feedback on the dorsiflexion force. **b** Sequence of a trial. Participants increased the dorsiflexion force to 8% MVC at the beginning of a trial, and the mental tasks were performed during the task phase. **c** Overview of the *trial* and *task* correlation analyses. The cumulative spike train (CST) of spinal motor neurons was extracted from HD-EMG signals. The EEG and CST trial variances were calculated over the task phase, yielding band-power values which were then correlated across individual trials and task averages

Spinal motor neurons (MNs) within the same motor pool receive common synaptic input [1]. This common synaptic input can be revealed by summing up the spiking activities of MNs, forming the cumulative spike train (CST). The CST reflects the neural drive transmitted to the innervated muscle [1]. Interestingly, the CST, and so the common synaptic input, carries oscillations at least up to 75 Hz [2], but only frequencies below 10 Hz are transduced effectively into muscle force changes. Oscillations with frequencies at about 10 Hz and higher are filtered out by spinal networks [3, 4] and the low-pass filtering characteristics of the muscle [5–7]. Oscillatory activities found in the beta band (13–30 Hz) during tonic muscle contractions and in the gamma band (30–70 Hz) during dynamic muscle contractions are at least partly cortical in origin, as shown by connectivity analyses like cortico-muscular coherence (CMC) [8–14]. The functional interpretation of these high-frequency oscillations is unclear as they do not directly affect muscle force. They may serve as a probing signal to the periphery, facilitate a steady motor output, or relate to motor readiness [15–17]. Another hypothesis, which is not mutually exclusive, is that some components of the CST in the beta or gamma band represent cortical oscillations that do not have a particular function for ongoing motor control but nonetheless propagate downstream to MNs. These oscillations could be carried by corticomotoneuronal pathways [14, 18–20]. Since the cortical oscillations in the beta and gamma bands are not within the effective musculoskeletal bandwidth, they would not interfere with the force-generating neural drive and would thus be functionally separable from it. If the latter hypothesis is true, *modulations* of such cortical oscillations—that propagate downstream to MNs but do not contribute to ongoing motor control—should be visible in the CST of the respective MNs and independent of ongoing force output. To test this hypothesis, we investigated whether power modulations of cortical oscillations induced by mental tasks distinct from an ongoing motor task are paralleled by corresponding modulations in the CST of spinal MNs. For that, we asked human participants to perform various mental tasks (foot motor imagery, hand motor imagery, mental arithmetic, and no task) while simultaneously executing a static isometric dorsiflexion of the right foot (see Fig. 1a).

The selected mental tasks span a continuum of expected sensorimotor engagement: foot motor imagery engages neural representations closely overlapping with the executed dorsiflexion [21, 22], hand motor imagery is somatotopically distinct from the executing effector [21, 22], and mental arithmetic primarily involves attentional and executive networks [23]. All three tasks were expected to directly or indirectly modulate cortical oscillatory activity over the sensorimotor cortex [21, 22, 24–26] and thus could affect spinal output.

We hypothesised that power modulations of cortical oscillations induced by these mental tasks propagate downstream to MNs, are detectable in their CST, and are independent of ongoing force control. The CST activity was obtained by decomposing MN activity from high-density electromyography (HD-EMG) signals recorded non-invasively from the right tibialis anterior (TA) muscle [27]. We calculated CST and electroencephalography (EEG) bandpower across frequency bands, and analysed CST-EEG bandpower correlations across *trials* and *mental tasks* to identify transmitted oscillations (see Fig. 1a, c).

Mental-task induced cortical oscillations that propagate to spinal motor neurons could potentially provide control signals for neural interface applications, analogous to oscillation-based brain-computer interfaces (BCIs) [28]. Applications could include motor neuroprostheses for restoring lost function [29, 30] or movement augmentation [31]. To assess the viability of such a control approach, we evaluated mental task discriminability from CST bandpower using signal-to-noise ratio (SNR) analysis and single-trial classification.

## Methods

### Participants

Fifteen participants were recruited, including 11 men and 4 women. The participants were between 21 and 47 years old (28.3 ± 6.1 years; mean ± std. dev.). Recruitment was independent of any previous experience with electrophysiology experiments and handedness. The study was approved by the ethics committee of the University of Freiburg (approval number 21-1388-1), and written informed consent was obtained from the participants.

### Experimental paradigm

The participants were seated on a comfortable chair with their arms placed on armrests. Their right leg was fastened to a foot ankle dynamometer (NEG1, OT Bioelettronica SRL, Italy). A computer screen placed in front provided force feedback and instructions to the participants. We used a self-developed software package for this purpose (YAGA^1^). We asked participants to perform an isometric maximum voluntary contraction (MVC) with their right foot (dorsiflexion). This was done to allow the presentation of the relative force value later in the recording session. Participants performed three MVCs for 5 s each. The maximum force value among the three contractions was selected as the MVC force value. The participants were then familiarised with the force feedback by tracking a trapezoidal force profile with their right foot five times (10 s ramp up, 5 s hold at 10% MVC, 10 s ramp down). They were able to see the relative dorsiflexion force on the computer screen along with the target force. After that, the participants tracked a similar trapezoidal force profile four times but with a hold phase of 30 s.

Next, we instructed participants to perform a static isometric dorsiflexion with their right foot at 8% MVC while simultaneously performing one of four mental tasks, see Fig. 1a. The four mental tasks were: motor imagery (MI) of right foot dorsi/plantarflexion (*foot MI* task), motor imagery of both hands opening/closing (*hand MI* task), continuously subtracting the number 3 from a random number displayed on the computer screen (*math* task), and no additional task (*no task* task). For the two MI tasks, the participants were instructed to perform kinesthetic MI [22] of repetitive 0.5 Hz movements. The detailed sequence of a *trial* is shown in Fig. 1b. The participants started the isometric dorsiflexion at trial start and maintained the target force until the end of the trial. During the trial, the force feedback from the isometric dorsiflexion was provided by a ball moving on the computer screen’s vertical axis. At 5 s, the task cue was presented, indicating the mental task to perform. Participants performed the requested task for 5 s until the end of the trial. No feedback was given on the execution of the mental tasks. The time interval from 6 s to 9.5 s is referred to as the *task phase*. A *run* comprised 24 consecutive trials with a random inter-trial interval of 4 to 6 s. We recorded 15 runs with breaks of at least 1 min between runs. The first run was a familiarisation run to ensure proper task execution. In total, we recorded effectively 336 trials, i.e., 84 trials per task.

In the end, we again asked the participants to track four trapezoidal force profiles with a 30 s hold phase (data from trapezoidal force tracking tasks were not further used in this manuscript).

### Recording

We recorded electrical activity from the tibialis anterior muscle using an HD-EMG grid electrode (GR08MM1305, OT Bioelettronica SRL, Italy). The grid contained 64 monopolar electrodes with 13 mm inter-electrode distance and was placed on the muscle belly aligned with the fibre direction. EMG reference and EMG ground electrodes were placed on the right and left ankle, respectively. Furthermore, we recorded bipolar EMG signals from the right gastrocnemius muscle and extensor digitorum and flexor digitorum muscles of both arms. The force signal was measured with a foot-ankle dynamometer connected to a pre-amplifier (forza, OT Bioelettronica SRL, Italy). The EMG and force signals were recorded using a biosignal amplifier (Quattrocento, OT Bioelettronica SRL, Italy) with a sampling rate of 2048 Hz. The EMG signals were filtered using a 0.7 to 900 Hz hardware bandpass filter. We recorded electrical brain activity with a 61-channel EEG montage of active Ag/AgCl electrodes covering frontal, central, parietal and temporal areas. The EEG ground electrode was placed on Fpz; a dedicated physical reference electrode was not employed, i.e., the acquisition was reference-free. Additionally, we recorded electrooculography (EOG) signals with three electrodes placed above the nasion and below the outer canthi of both eyes. The EEG and EOG signals were recorded with a second biosignal amplifier (actiCHamp, Brain Products GmbH, Germany) and sampled at 2000 Hz with a 530 Hz anti-aliasing filter. The signals from both biosignal amplifiers were recorded and synchronised using Lab Streaming Layer (LSL)^2^. To access the data from the Quattrocento biosignal amplifier, we developed a custom LSL application^3^.

### Preprocessing

The signals were processed with Matlab R2023b (MathWorks, USA) and the external toolboxes BioSig 3.7.6 [32] and EEGLAB v2022.0 [33]. To compute the relative force, we filtered the force signal using a median filter with a window length of 31 samples and normalised it to the participants’ MVC force levels. We referenced the EEG and EOG signals to AFz. Next, the (HD-)EMG, EEG, EOG and force signals were downsampled using a common time base to 1024 Hz for easier processing in terms of memory consumption and computation time. A 400 Hz anti-aliasing filter was applied before downsampling (4^th^-order zero-phase Butterworth filter). To suppress line interference, we applied a notch filter at 50 Hz and at the first two harmonics to the (HD-)EMG, EEG and EOG signals. Finally, we filtered the EEG/EOG data from 2 Hz to 150 Hz with a band-pass filter, and the force signal with a 10 Hz low-pass filter (4^th^-order Butterworth) to suppress non-physiological signal components.

We visually inspected HD-EMG, EEG and EOG signals and excluded noisy or bad channels. Possible stationary artefacts in EEG signals were removed with an independent component analysis (ICA) [33, 34]. We visually inspected ICs and removed components representing typical artefacts such as eye blinks, eye movements or muscle activity. Trials with EEG values exceeding $\pm \,{250}\,\upmu \text {V}$ were excluded from the ICA and the inspection of ICs.

Finally, we excluded trials with transient artefacts in the *task phase* from the subsequent analyses. For the analyses relying solely on HD-EMG signals, we excluded trials with relative force values below 6% or above 10% MVC. Next, we excluded trials with abnormal force mean values or force standard deviations in the task phase. Moreover, we excluded trials with abnormal task-phase HD-EMG power values (wrt. the maximum across channels). Abnormal trials were defined as trials in which the respective statistic (i.e., force mean values, force std. dev., and HD-EMG power) deviated more than 7.413 times the median absolute deviation (MAD) from the median over trials. MAD is a robust alternative to the standard deviation, and the threshold corresponds to 5 times the standard deviation for normally distributed data [35]. In total, this led to an exclusion of 0% to 29% of the trials based on force and HD-EMG signals (6% ± 8%; mean ± std. dev. over participants). For the analyses relying solely on EEG data, we excluded trials with EEG amplitude values outside ± ${75}\,\upmu \text {V}$, or abnormal task-phase EEG power values (wrt. the maximum across channels) using a threshold of 7.413 times the MAD. Furthermore, we computed joint probability and Kurtosis of the EEG amplitude values for each trial and channel using EEGlab [33]. We excluded trials where either statistic exceeded a threshold of 5 times the standard deviation from the mean across trials for that statistic for any channel. In total, this led to an exclusion of 3% to 11% of the trials based on EEG signals (6% ± 3%; mean ± std. dev.). All criteria were combined for analyses relying on HD-EMG and EEG signals, resulting in the exclusion of 5% to 32% of the trials (12% ± 7%; mean ± std. dev.).

### EMG signal decomposition and cumulative spike train

A validated MN decoder from Negro et al. [27] was used to extract spiking activity of MNs from HD-EMG signals. The MN decoder was trained with HD-EMG data from all trials within the time interval of 5 s to 9.5 s relative to trial start. This decoder uses a blind source separation approach to decompose an HD-EMG signal into spike trains of MNs. To do this, the decoder algorithm extends the multi-channel HD-EMG signal with additional channels comprising time-lagged versions of the original HD-EMG signal, whitens the extended signal, and finds sparse source signals with a fixed-point algorithm [36, 37]. Typically, for signals sampled at around 2 kHz, the signal is extended with 16 successively increasing time-lags. Since we downsampled the signals to 1024 Hz, we selected 8 time lags to effectively cover the same time period. The fixed-point algorithm is followed by an iterative refinement step that uses a clustering approach to separate spikes from noise. A source signal was eventually accepted if it had a silhouette measure (SIL) [27] higher than 0.85. Due to the extension with time-lags, the decoder algorithm can find duplicate spike trains which are generated by a single MN but shifted by a constant time (the same spikes are detected at different time-lags). To detect duplicate spike trains, we applied agglomerative hierarchical clustering. We considered spike trains to be in the same cluster, i.e., belonging to the same MN, if they shared more than 5% of identical spikes. Here, we allowed one spike train to be shifted up to ± 32 samples relative to another to compensate for a possible constant time shift between spike trains. After compensating for this potential time shift, we considered spikes from two spike trains to be identical if they differed by a maximum of one sample.

We then kept only MNs (1) which had an average spike rate of more than 5 Hz, (2) where the standard deviation of the average firing rate across trials was less than 1.5 Hz (after detrending the spike rate with a moving average over 50 trials), and (3) where the trial-averaged coefficient of variation (CoV) of the inter-spike interval was less than 0.5. We considered only the task phase for all three criteria. This yielded MN counts between 2 and 35 (15.9 ± 10.5; mean ± std. dev. over participants).

The CST was eventually obtained by summing the individual spike trains of the MNs.

### Power comodulation analysis

We analysed whether oscillations in the CST were comodulated with brain signals (see Fig. 1c). To do this, Source Power Comodulation (SPoC) [38] was used to find a linear projection of EEG channels that maximises covariance with CST in bandpower. In other words, SPoC found a spatial filter that extracted an EEG source, whose bandpower had maximal covariance with the CST bandpower.

First, we applied a filter bank from 5 to 74 Hz to the CST. The filter bank consisted of 5 Hz wide bands, separated by 1 Hz steps (i.e., 5-9, 6-10, ..., 70-74 Hz; 4th-order zero-phase Butterworth). We calculated the variance over the task phase to obtain the bandpower $CSTBP_{f,i}$:

$$\begin{aligned} CSTBP_{f,i} = \mathop {\textrm{var}}\limits \left( CST_{f,i}(t) \right) \end{aligned}$$

where $CST_{f,i}(t)$ is the $i^{th}$ trial of the bandpass filtered CST of frequency band *f*, and *t* indexes the time within the task phase of the trial. Subsequently, the signals were detrended to compensate for possible non-stationarities during a recording session. For that, we subtracted the moving median using a window length of 50 trials and obtained the detrended bandpower:

$$\begin{array}{l}\!\!CSTBP^{detrend}_{f,i} = CSTBP_{f,i} - \mathop {\textrm{movmedian}}\limits \left( CSTBP_{f,i} \right) \end{array}$$

Potential *task-dependent* changes in force output could have confounded CST bandpower. To remove possible force-related confounders, we computed the mean value and standard deviation of the force as well as the CST rate during the task phase. We then trained for each frequency band a multiple linear regression model with force mean, force standard deviation, and CST rate as predictor variables, and $CSTBP^{detrend}_{f,i}$ as response variable. Only the residuals $CSTBP^{res}_{f,i}$ were then used for further analysis. The residuals corresponded to the original CST bandpower features but with the linear effects of force and CST spike rate removed. Next, we applied the same filter bank to the EEG signal and applied SPoC separately for each frequency band. The SPoC implementation provided in [39] with shrinkage [40, 41] was used for that. We calculated the variance over the task phase of the EEG channel projection found by SPoC (only the first SPoC component was used):

$$\begin{aligned} SPOC_{f,i} = \mathop {\textrm{var}}\limits \left( \sum _{c} EEG_{c,f,i}(t) \cdot w_{c,f} \right) \end{aligned}$$

where $EEG_{c,f,i}$ is the EEG signal with *c* indexing the EEG channel, and $w_{c,f}$ the weight found by SPoC. SPoC found the weights $w_{.,f}$ such that the covariance over trials between $SPOC_{f,i}$ and $CSTBP^{res}_{f,i}$ was maximized. Importantly, $SPOC_{f,i}$ was found using a 10-fold cross-validation to prevent overfitting of the SPoC weights. Thus, the computation of the SPoC weights and their application was performed on separate trial sets obtained by cross-validation. Subsequently, we calculated a robust average of CST and SPoC trials over the mental tasks:

$$\begin{aligned} \begin{aligned} \widetilde{CSTBP}_{f,task}&= \mathop {\textrm{median}}\limits _{i \in S_{task}} \left( CSTBP^{res}_{f,i} \right) \\ \widetilde{SPOC}_{f,task}&= \mathop {\textrm{median}}\limits _{i \in S_{task}} \left( SPOC_{f,i} \right) \\ \end{aligned} \end{aligned}$$

where $S_{task}$ is the set of trial indices belonging to *task*.

Eventually, the correlation over individual trials (*trial* correlation) and over tasks (*task* correlation) was computed. To this end, we computed the *Kendall rank correlation coefficient* $\tau$ between CST and SPoC variances for each frequency band to identify comodulation. $\tau$ is a robust correlation measure as it is based on rank correlation. The *trial* correlation was calculated after removing the effect of the task from CST and SPoC trials:

$$\begin{aligned} \begin{aligned} CSTBP^{*}_{f,i}&= CSTBP^{res}_{f,i} - \widetilde{CSTBP}_{f,task} \\ SPOC^{*}_{f,i}&= SPOC_{f,i} - \widetilde{SPOC}_{f,task} \\ \tau _{trials,f}&= \mathop {\textrm{corr}}\limits _{i} \left( CSTBP^{*}_{f,i},\, SPOC^{*}_{f,i} \right) \\ \end{aligned} \end{aligned}$$

where *task* is the performed task in trial *i* and *corr* refers to the Kendall rank correlation coefficient. The *task* correlation was computed as the correlation over task averages:

$$\begin{aligned} \begin{aligned} \tau _{tasks,f}&= \mathop {\textrm{corr}}\limits _{task} \left( \widetilde{CSTBP}_{f,task},\, \widetilde{SPOC}_{f,task} \right) \\ \end{aligned} \end{aligned}$$

It should be noted that $\tau _{tasks,f}$ was computed using only four data points (mental tasks) per participant. This limited sample size resulted in a high variance of $\tau _{tasks,f},$ which was addressed with statistical tests conducted across participants. We computed $\tau _{tasks,f}$ using the Matlab implementation of Kendall’s $\tau$ coefficient, which includes an adjustment for ties, known as Kendall’s $\tau _b$ [42]. $\tau _b$ is identical to the originally proposed Kendall’s $\tau$ coefficient ($\tau _a$) in the absence of ties in any variable. $\tau _a$ is known to be an *unbiased* estimator for any bivariate distribution [42]. Furthermore, for independent continuous random variables, $E[\tau _a]=0$ [42]. Since CST or SPoC trials did not have any ties, i.e., no CST or SPoC trials had exactly the same power values, our estimation of Kendall’s $\tau$ coefficient was unbiased. Thus, the small sample size did not introduce any systematic error. In additional confounder analyses, we also calculated $\tau _{tasks,f}$ between EEG and force-related parameters (i.e., force mean, force standard deviation, and CST rate). In these analyses ties were present and we removed any potential bias with non-parametric bootstrap sampling [43] using 5000 repetitions.

The trial and task correlation coefficients were computed for each participant and frequency band. We performed group-level analyses on (1) the correlation coefficients of individual frequency bands and on (2) the correlation coefficients averaged across frequency bands covering 15 to 45 Hz (*band-averaged* correlations). This frequency range was chosen to cover the beta and low-gamma band of potentially transmitted brain oscillations. Furthermore, (3) we averaged for each participant the correlation coefficients of the 5 neighbouring frequency bands centred at the frequency with the highest common synaptic input, as determined by intramuscular coherence (*IMC-aligned* correlations). For the calculation of the intramuscular coherence (IMC), the MNs were first randomly split into two equally sized sets. We computed the CSTs of both sets [44] and applied a short-time Fourier transform (STFT) to the set-CSTs using a 1 s Hamming window. Next, we computed the power spectra of both set-CSTs and their cross-power spectrum, averaged them over the task phases, and calculated the coherence [45] between both MN sets. The random MN splitting was repeated a total of 500 times, and the coherences were eventually averaged over all random splits to obtain the IMC. We determined the peak frequency of the IMC between 15 and 45 Hz in order to find the frequency with the largest common synaptic input. The peak frequency was defined here as the frequency of the peak with the largest prominence [46].

### Discriminability analysis

We analysed whether the mental tasks modulated the power of the CST oscillations. To this end, the discriminability of the CST band-power $CSTBP^{res}_{f,i}$ was evaluated with SNR analysis and single-trial classification.

#### SNR analysis

The SNR at each frequency band during the task phase was quantified. For that, we defined the signal as *task-dependent band-power variability* (i.e., variance of task-averaged CST band-power):

$$\begin{aligned} P_{signal,f} = \mathop {\textrm{var}}\limits _{task} \left( \mathop {\textrm{mean}}\limits _{i \in S_{task}} \left( CSTBP^{res}_{f,i} \right) \right) \end{aligned}$$

where $S_{task}$ is the set of trial indices belonging to *task*. We defined the noise as *task-independent band-power variability* (i.e., variance of CST band-power across trials after removing the average task band-power):

$$\begin{aligned} P_{noise,f} = \mathop {\textrm{var}}\limits _{i} \left( CSTBP^{res}_{f,i} - \mathop {\textrm{mean}}\limits _{j \in S_{task}} \left( CSTBP^{res}_{f,j} \right) \right) \end{aligned}$$

where *task* is the performed task in trial *i*. As the variance operator involves calculating the mean, we used the mean rather than the median to determine the task-averaged CST bandpower values for consistency. We did not use MAD as an alternative to the variance in the calculation of the SNR due to the limited number of data points (i.e., 4 tasks). The SNR was eventually calculated as:

$$\begin{aligned} SNR_{f} = \frac{P_{signal,f}}{P_{noise,f}} - bias_f \end{aligned}$$

where the bias term $bias_{f}$ was determined with non-parametric bootstrap sampling [43] using 5000 repetitions. We computed the SNR for the frequency bands 5-9, 6-10, ..., 70-74 Hz. Additionally, we calculated the average SNR over the frequency range of 15 to 45 Hz (*band-averaged* SNR), and the average SNR over the 5 adjacent frequency bands centred at the IMC peak (*IMC-aligned* SNR). A baseline SNR $SNR_{baseline,f}$ was computed using the same procedure as described before, but using the time interval 2.5 to 5 s relative to the trial start, where no task information was available yet. Furthermore, we computed the SNR using the central EEG electrode Cz. The electrode Cz was chosen as it covers the foot area of the motor cortex and has been reported to yield the highest corticomuscular coupling with the tibialis anterior muscle [13, 47]. For this analysis, a Laplace spatial filter was applied to extract brain activity predominantly from the motor cortex [48, 49].

#### Single-trial classification

We quantified the single-trial discriminability of CST bandpower features (*CST-FB*) with a shrinkage linear discriminant analysis (sLDA classifier) [50, 51]. The shrinkage parameter was computed analytically using the method of Ledoit and Wolf [40, 41]. The features were the bandpower values $CSTBP^{res}_{f,i}$ computed with a filter bank as in section “Power comodulation analysis” but with a frequency range covering beta and low-gamma bands in 2 Hz steps (i.e., 15–19, 17–21, ..., 41–45 Hz). Additionally, we classified CST bandpower features (*CST-IMC*) obtained only from the participant-specific IMC peaks and the 4 neighbouring frequency bands (stepsize 1 Hz). Furthermore, we evaluated the discriminability of EEG signals and extracted bandpower features from all 61 monopolar EEG channels using the same filter bank as the CST-FB features. We were only interested in EEG frequencies which could potentially leak down to MNs. We therefore omitted the mu-band (8–13 Hz), which is often task-discriminative, as signals around 10 Hz are attenuated during downstream propagation [1]. We also quantified the discriminability of potential confounders. This was done by calculating the mean and standard deviation of the force, as well as the CST rate, within the task phase (*FORCE-MEAN,* *FORCE-STD* and *CST-RATE*). We then computed the classification accuracies yielded by the features using an sLDA classifier and a $10 \times 10$ fold cross-validation.

The agreement of classifier predictions from CST-FB and EEG-FB features was evaluated with the *uncertainty coefficient* [52]. The uncertainty coefficient *U* is a measure of nominal association derived from information entropy, ranging between 0 and 1:

$$\begin{aligned} U(X \vert Y) = \frac{I(X; Y)}{H(X)} \end{aligned}$$

with *I*(*X*; *Y*) being the mutual information between *X* and *Y*, and *H*(*X*) the entropy of *X*. $U(X \vert Y)$ is not symmetric with respect to *X* and *Y* and a symmetric uncertainty coefficient can be obtained as [52]:

$$\begin{aligned} U(X, Y) = \frac{H(X)U(X \vert Y) + H(Y)U(Y \vert X)}{H(X) + H(Y)} \end{aligned}$$

For each participant, we first computed the task predictions separately from CST-FB and EEG-FB features using a $10 \times 10$ fold cross-validation with common train and testsets. Subsequently, we computed *U*(*X*, *Y*) across all testsets of a cross-validation repetition (i.e., a reshuffling of trials). We then removed the bias with non-parametric bootstrap sampling [43] using 1000 repetitions, and eventually averaged *U*(*X*, *Y*) over all 10 cross-validation repetitions.

### Independence of task effects on CST bandpower from force

To test whether the mental tasks modulated CST bandpower independently of force, we fitted a linear model on the trial level for each participant. CST bandpower was computed as the average across the 5 frequency bands centred at the participant-specific IMC peak frequency. The four mental task conditions were encoded using three binary dummy variables $\text {task}_1,$ $\text {task}_2,$ $\text {task}_3.$ The *no task* condition was encoded by setting all dummy variables to zero; each remaining task was encoded by setting exactly one dummy variable to one.

The model predicted CST bandpower from force mean and task:

$$\begin{array}{c} \text {CST BP} = \alpha _0 + \alpha _1 \cdot \text {force mean} + \alpha _{2} \cdot \text {task}_1 \\ \hfill + \alpha _{3} \cdot \text {task}_2 + \alpha _{4} \cdot \text {task}_3 \end{array}$$

To quantify whether task contributed explanatory power beyond force, we compared the full model against the nested model obtained by removing the set of three task dummy variables. We computed the change in adjusted $R^2$ ($\Delta R^2_{adj}$), defined as the adjusted $R^2$ of the full model minus the adjusted $R^2$ of the nested model [53, Section 3.3.2]. The adjusted $R^2$ corrects for the number of predictors in the model and was calculated as $1 - (1 - R^2) \cdot \frac{n - 1}{n - p}$ [54]. A positive $\Delta R^2_{adj}$ indicates that task explained variance in CST bandpower not accounted for by force.

Additionally, to rule out that a significant task effect in the model above could arise from a collider structure (Task $\rightarrow$ Force $\leftarrow$ CST BP), in which conditioning on force creates a spurious association between task and CST bandpower, we tested whether task and CST bandpower were marginally associated. For this, we fitted the reduced model $\mathrm{CST}\: \mathrm{BP} = \alpha _0\: +\: \alpha _{2}\: \cdot\: \text {task}_1 + \alpha _{3} \: \cdot\: \text {task}_2 + \alpha _{4}\: \cdot \: \text {task}_3$ without force as a predictor and computed its adjusted $R^2$. Since the nested model in this case contains only the intercept, whose adjusted $R^2$ is zero, the adjusted $R^2$ of the reduced model directly quantifies the marginal association without requiring a difference. A significant marginal association between task and CST bandpower would rule out the collider structure, as in a collider the two parent variables are marginally independent.

The per-participant $\Delta R^2_{adj}$ values and the marginal adjusted $R^2$ values were tested against zero using a two-sided Wilcoxon signed-rank test, corrected for multiple comparisons using the Benjamini & Hochberg procedure [55] ($q = 0.05$). A significant task effect after controlling for force, combined with a significant marginal association, would indicate that the task effect on CST bandpower is not fully accounted for by force and is not an artefact of conditioning on a collider.

### Statistics

For comparisons involving one or two samples, non-parametric tests were employed. Specifically, two-sided Wilcoxon signed-rank tests were used except for the symmetric uncertainty coefficient *U*(*X*, *Y*). The Wilcoxon signed-rank test tests the null hypothesis that a sample or the difference between two paired samples comes from a symmetric distribution with a median of zero. The significance level was set to $\alpha =0.05$. For multiple comparisons, the false discovery rate was controlled at $q=0.05$ with the Benjamini & Hochberg procedure [55], and we report Benjamini & Hochberg adjusted p-values ($p_{adj}$) [56]. Adjusted p-values reported in the same paragraph in section “Results” were considered as a set to which the Benjamini & Hochberg method was applied. For the symmetric uncertainty coefficient *U*(*X*, *Y*), statistical significance was evaluated at the participant level using a one-sided permutation test [57, 58], where the CST-FB and EEG-FB labels were permuted across all testsets within a cross-validation repetition. We sampled $N=10000$ symmetric uncertainty coefficients from the permutation distribution. The p-values were calculated as $p=(b+1)/(N+1)$, where *b* is the number of symmetric uncertainty coefficients from the permutation distribution greater than or equal to the observed symmetric uncertainty coefficient [59]. A one-sided test is justified as any value of *U*(*X*, *Y*) smaller than the permutation distribution is to be considered a random fluctuation. We used repeated measures ANOVA for comparisons involving more than 2 samples. If the sphericity assumption was violated (Mauchly’s test), a Greenhouse-Geisser (GG) correction was applied. Post-hoc analysis was performed with a Tukey-Kramer test. Confidence intervals of statistics based on one or two samples were computed at a level of 95% using bootstrapping with the bias-corrected and accelerated percentile method [60].

## Results

### Representative motor neuron activity

**Fig. 2:**
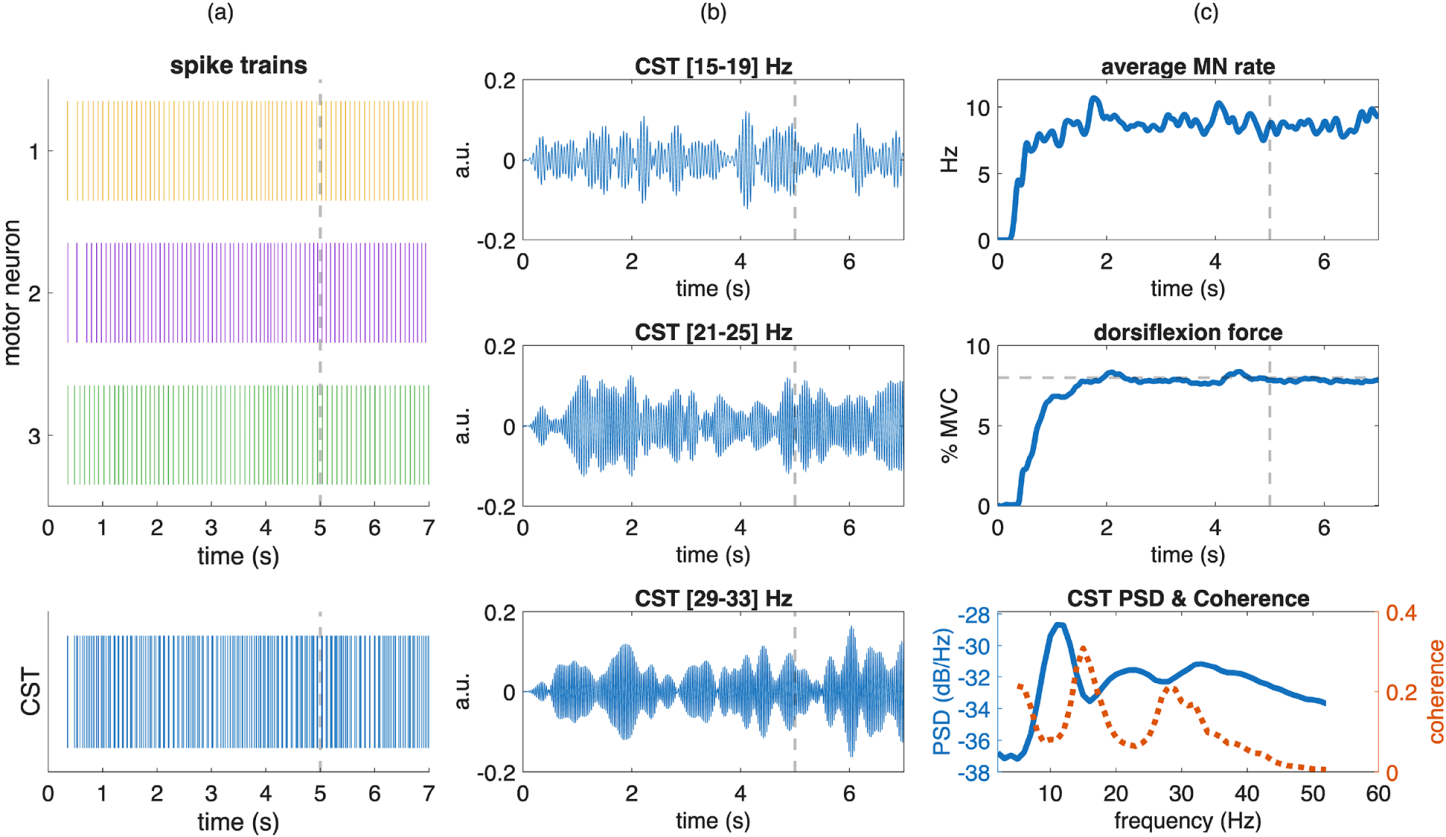
Exemplary data from a representative trial. Trial onset occurred at $t=0\,{s}$, the task cue was presented at $t=5\,{s}$ (dashed vertical line). **Column** **a** Selected motor neuron spike trains and their cumulative spike train (CST). **Column b** Bandpass-filtered CST across three representative frequency bands. **Column** **c** Average firing rate of the MNs and dorsiflexion force over trial time. The dashed horizontal line represents the target force level of 8 % MVC. The bottom plot shows the power spectral density (PSD; blue) and the coherence (red) averaged across all trials

Figure 2 shows representative motor neuron activity from a single trial and participant. Individual motor neurons were decomposed from HD-EMG recordings and summed to form the cumulative spike train (CST) (Fig. 2a). The CST was analysed using bandpass filtering across multiple frequency bands, with three representative bands shown in Fig. 2b. Figure 2c shows the average firing rate of the MUs alongside the dorsiflexion force. The average MN firing rate was estimated from the CST using Gaussian kernel density estimation with a 50 ms bandwidth [61] and divided by the number of decoded motor neurons. The initial increase in both rate and force reflects the force ramp-up to the target level at trial onset. The trial-averaged power spectral density in Fig. 2c reveals frequency peaks above 10 Hz.

### Power comodulation between EEG and motor neuron activities

We calculated the Kendall rank correlation coefficient $\tau$ between the bandpowers of CST and EEG to reveal a potential power comodulation between MN and brain activity. Bandpower was calculated from 5 Hz wide bands, separated by 1 Hz steps (i.e., 5-9, 6-10, ..., 70-74 Hz). EEG signals were first mapped to a one-dimensional space using a data-driven spatial filter (SPoC [38]) before EEG bandpower was calculated.

#### Correlation of frequency bands

**Fig. 3:**
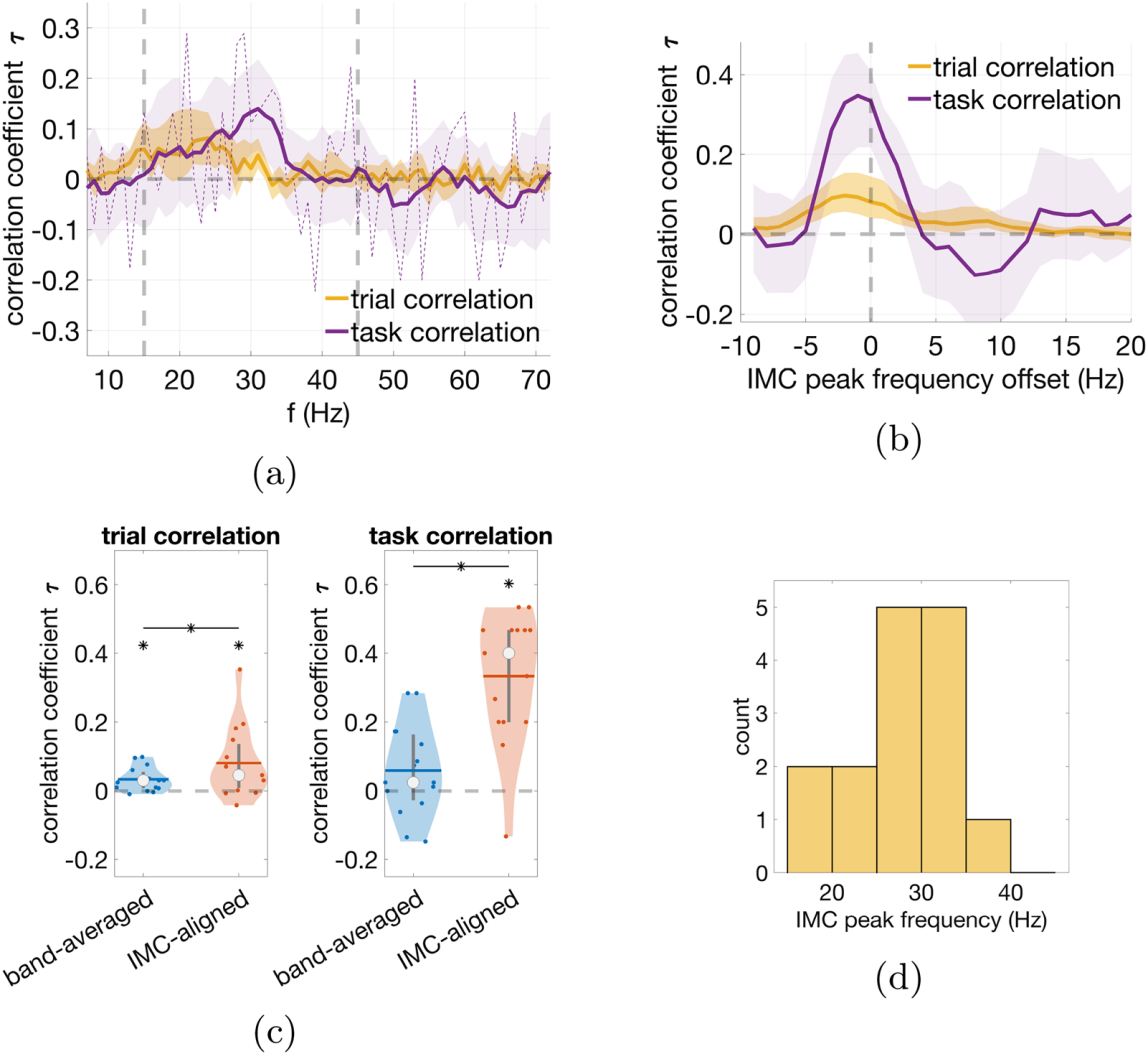
CST-EEG correlation results. **a** Trial and task correlations as a function of frequency. Shown are group averages and their 95% confidence intervals. The task correlations were smoothed by a moving average filter across 10 frequency bands; the unsmoothed task correlations are shown by a purple dashed line. The vertical dashed lines mark the frequency range used for calculating the band-averaged correlations. **b** IMC-aligned trial and task correlations over IMC peak frequency offsets. Shown are group averages and their 95% confidence intervals. **c** Band-averaged and IMC-aligned trial and task correlations of the individual participants. The group medians are marked with a circle; the group mean values are marked with a horizontal line; the vertical grey lines indicate the interquartile ranges. A star marks statistically significant differences between groups or against 0 (two-sided Wilcoxon signed-rank test, $q=0.05$). **d** Distribution of the found IMC peak frequencies

The correlation between CST and EEG bandpower was computed (1) across all *trials* irrespective of the task as *trial correlation* ($\tau _{trials}$), and (2) across *tasks* using the median power values of each task as *task correlation* ($\tau _{tasks}$). Figure 3a shows the group average of the trial and task correlations and their 95% confidence intervals as a function of frequency. The task correlations were noisy due to the small number of data points per participant (4 data points or tasks). For visualisation purposes, we therefore smoothed the task correlations before averaging over participants with a moving average comprising 10 frequency bands. The group average of the trial correlations peaked in the beta band at 24 Hz with a value of 0.08. The group average of the smoothed task correlations showed a peak at 31 Hz with a value of 0.14. Both trial and task correlations displayed increased correlations in the beta range from approximately 15 to 25 Hz. Moreover, the task correlation was increased in the low-gamma band around 30 Hz. The positive *task correlations* indicate that the mental tasks modulated the EEG and CST bandpower signals in the same direction. In addition, the prominent positive *trial correlations* suggest that there were *spontaneous* trial-by-trial correlations between EEG and CST bandpower signals that were not caused by the mental tasks.

#### Band-averaged and IMC-aligned correlations

The correlations were then averaged over the beta and low-gamma bands (15–45 Hz), which we refer to as *band-averaged* correlation. In addition, we determined the participant-specific frequency with the largest common synaptic input, as measured by IMC, and averaged the correlations around the IMC peak (*IMC-aligned* correlation).

The *band-averaged* and *IMC-aligned* correlations are shown in Fig. 3c and in Table 1. A two-sided Wilcoxon signed-rank test was used to test for significant differences, and p-values were adjusted for multiple comparisons. Both band-averaged and IMC-aligned *trial* correlations were significantly greater than 0 ($W=113,$ $p_{adj}=0.002$, and $W=109$, $p_{adj}=0.005$, respectively). Furthermore, the IMC-aligned trial correlation was significantly larger than the band-averaged correlation ($W=23$, $p_{adj}=0.042$). The IMC-aligned *task* correlation was significantly larger than both 0 and the band-averaged correlation ($W=118.5$, $p_{adj}=0.001,$ and $W=0$, $p_{adj}={4\text {e}-04}$, respectively). In contrast, the band-averaged task correlation was not significantly larger than 0 ($W=88.5$, $p_{adj}=0.110$). Thus, the IMC alignment increased the trial and task correlations relative to the broadband average.

#### IMC peaks

The IMC peak was used to determine the frequency with the largest common synaptic input [7]. Figure 3d displays the histogram of the found peak frequencies. The majority of peaks were found in the upper-beta/lower-gamma band around 30 Hz, which is also the frequency range yielding the highest group task correlation. However, participants P5, P7, and P11, which exhibited greater task correlation, had IMC peak frequencies in the lower-beta band around 20 Hz (c.f. Fig. 5). Figure 3b shows trial and task correlations as a function of an offset to the IMC peak frequency. The highest average trial and task correlations were around an offset of -2 Hz and -1 Hz, respectively. Thus, the highest correlations were obtained around the IMC peak frequency, suggesting that the CST-EEG bandpower correlations are primarily due to oscillations carried by the common synaptic input.

#### Permutation distribution of IMC-aligned correlations

**Fig. 4:**
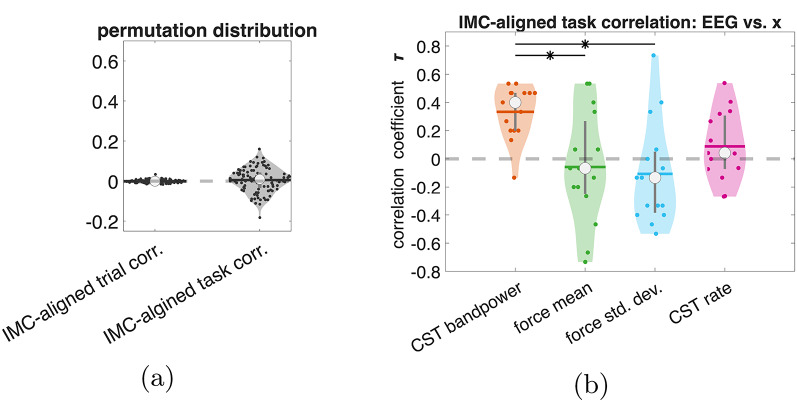
Bias and confounder correlation analyses. **a** Permutation distribution of IMC-aligned correlations. Individual data points are mean values across participants after trial shuffling. **b** IMC-aligned task correlations between SPoC-filtered EEG and CST bandpower or force-related parameters (force mean, force std. dev., CST rate). The group medians are marked with a circle; the group mean values are marked with a horizontal line; the vertical grey lines indicate the interquartile ranges. A star marks statistically significant differences between groups (Tukey-Kramer post-hoc test, $\alpha =0.05$)

To assess potential overfitting or bias, we created a permutation distribution by randomly shuffling trials and recomputing IMC-aligned trial and task correlations 100 times (Fig. 4a). The shuffled correlations had mean values near zero (trial correlation: $-0.001$; task correlation: 0.005), confirming the absence of bias or overfitting. The maximum shuffled values (trial correlation: 0.034; task correlation: 0.160) remained well below the observed sample means of 0.08 and 0.33, respectively (Table 1).

#### Dual-task subset correlations

The event-related desynchronization/synchronisation (ERD/S) analysis in section “EEG ERD/S Plots” revealed that only the dual-task conditions, i.e., *foot MI*, *hand MI*, and *math*, featured clear relative power decreases in the mu and beta bands, while the *no task* condition did not. The reported power comodulations could be solely due to bandpower differences between *no task* and *any* other dual-task, rather than bandpower differences within the dual-tasks. We therefore analysed whether there were significant power comodulations within the dual-task subset comprising *foot MI*, *hand MI*, and *math*. For this purpose, we used the same trained spatial SPoC filters but evaluated the power correlations using only the task subset. The obtained group-level correlations are shown in Table 2. The IMC-aligned task correlation was significantly larger than both 0 and the band-averaged correlation ($$W=116$$, $$p_{adj}=0.002$$, and $$W=6$$, $$p_{adj}=0.002,$$ respectively). The band-averaged correlation was also significantly larger than 0 $$(W=98.5$$, $$p_{adj}=0.032)$$. All tests were two-sided Wilcoxon signed-rank tests, and p-values were adjusted for multiple comparisons. This indicates that the observed power comodulations were not solely due to greater bandpower in *no task* compared to the other tasks, and that *foot MI*, *hand MI*, and *math* yielded common power changes in EEG and CST.

#### Task-dependent correlations with force

To assess whether EEG oscillations were comodulated with force-related parameters across mental tasks, we calculated task correlations between SPoC-filtered EEG and force-related parameters, namely force mean, force std. dev., and CST rate. We trained the SPoC filters using the individual force-related parameters as target signals, analogously to the procedure described in section “Power comodulation analysis”. The task correlations at the IMC peak (IMC-aligned) are shown in Fig. 4b, along with the task correlation for CST bandpower reported previously. At both the mean and median, CST bandpower showed the highest task correlation, while the force-related parameters were close to zero. We compared task correlations using a one-way repeated measures ANOVA with target signal type as the explanatory variable, and found a statistically significant difference between task correlations ($F(3,42)=6.860$, $p=0.001$). A Tukey-Kramer post-hoc test revealed statistically significant pairwise differences between CST bandpower and force mean ($p=0.044$, 95%-CI $=[0.01, 0.77]$), and CST bandpower and force std. dev. ($p=0.002$, 95%-CI $= [0.16, 0.72]$). CST bandpower and CST rate were not statistically significantly different ($p=0.063$, 95%-CI $=[0.01, 0.50]$).

These results support the interpretation that the observed CST-EEG bandpower correlations were mainly due to force-unrelated cortical oscillations propagating downstream rather than to task-dependent force output as a confounding factor.

**Table 1:** Group statistics of the Kendall rank correlation coefficient $\tau$ across trials and tasks

Correlation analysis		Mean value	Standard deviation	Median
trial correlation ($\tau _{trials}$)	band-averaged	0.03	0.03	0.03
	IMC-aligned	0.08	0.10	0.05
task correlation ($\tau _{tasks}$)	band-averaged	0.06	0.13	0.02
	IMC-aligned	0.33	0.19	0.40

**Table 2:** Group statistics of the Kendall rank correlation coefficient $\tau$ across the task subset. Only the mental tasks *foot MI*, *hand MI*, and *math* were considered

Correlation analysis		Mean value	Standard deviation	Median
task correlation ($\tau _{tasks}$)	band-averaged	0.09	0.14	0.06
	IMC-aligned	0.39	0.28	0.47

#### EEG spatial patterns

**Fig. 5:**
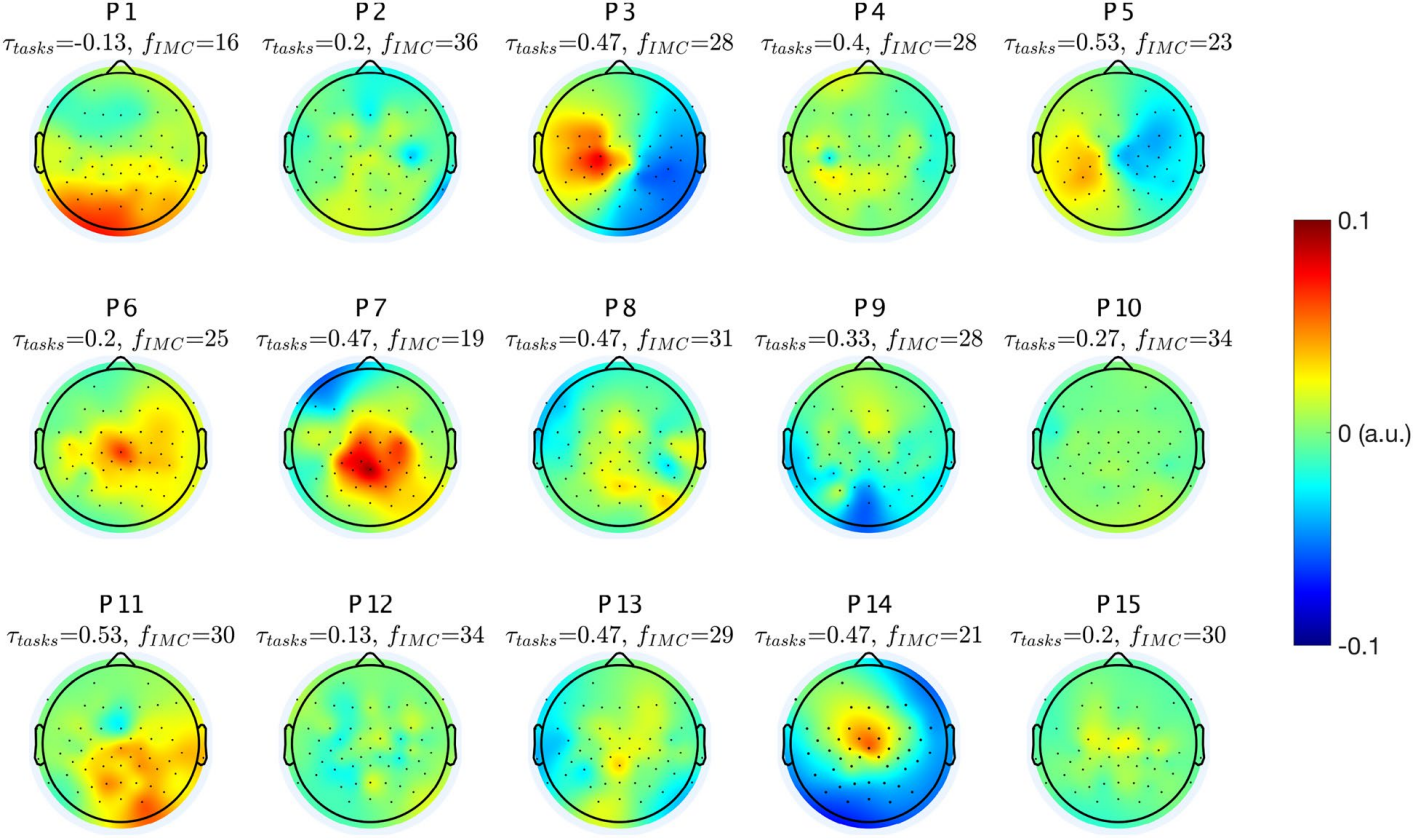
SPoC pattern of each participant and the IMC peak frequency $f_{IMC}$ in Hz with the respective task correlation coefficient $\tau _{tasks}$ at this frequency. SPoC patterns were computed to maximise covariance between EEG and CST bandpower across trials, without using any mental task class label information

SPoC finds a spatial filter that extracts a signal whose power has maximal covariance with a target variable [38]. One can find the corresponding *pattern* to this spatial filter. The pattern displays the source generating that signal. Figure 5 shows the channel-space SPoC patterns at the participant-specific IMC peaks and the associated task correlations $\tau _{tasks}$ and IMC peak frequencies. Participants P3, P5, P6, P7, and P14 displayed a pattern that suggests a dipole-like source located close to the foot area of the primary motor or somatosensory cortex. P1 and P11 displayed an occipital/parietal source, with only P11 having a greater correlation. Notably, P3, P5, P7 and P14 achieved relatively high correlation and classification accuracies (c.f. Table 4). Not all participants exhibited distinct and clear SPoC patterns. This is possibly due to the noisy nature of the EEG signal and the imperfect transmission of oscillations downstream to MNs, which affects the target signal used to train the SPoC model and thus the model itself.

### Signal-to-noise ratio (SNR) analysis

#### SNR of frequency bands

**Fig. 6:**
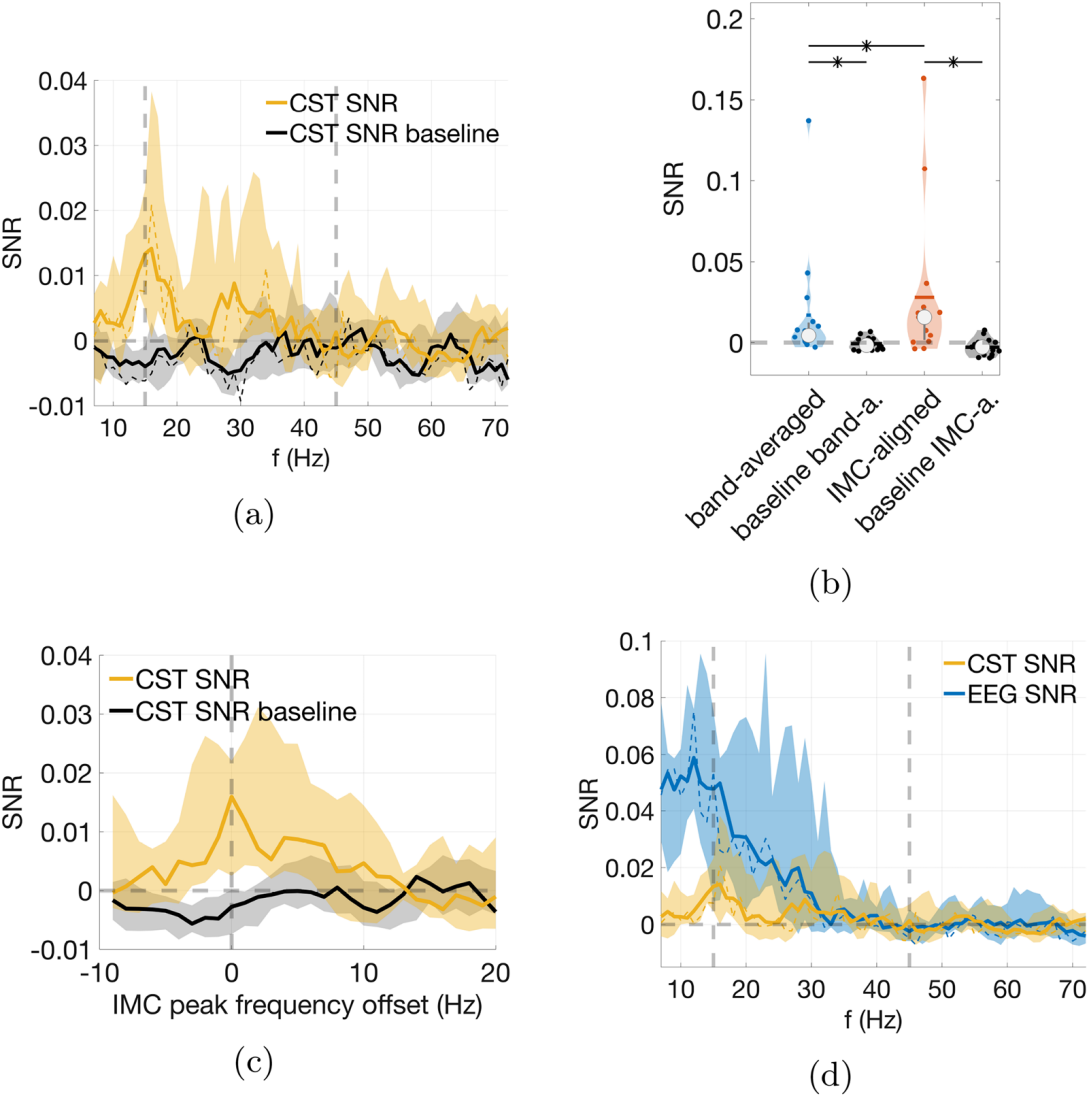
SNR results. **a**, **c**, and **d** show group medians and their 95% confidence intervals. **a** CST and baseline SNR as a function of frequency. SNR values were smoothed with a moving average filter using 5 frequency bands; the unsmoothed SNR values are shown as dashed lines. The vertical dashed lines mark the frequency range used to calculate the band-averaged SNR. **b** Band-averaged and IMC-aligned CST SNR of the individual participants with their corresponding baselines. The group medians are marked with a circle; the mean values are marked with a horizontal line; the vertical grey lines indicate the interquartile ranges. A star marks statistically significant differences (two-sided Wilcoxon signed-rank test, $q=0.05$). **c** IMC-aligned CST SNRs as a function of an offset to the IMC peak frequency. **d** CST SNR and EEG SNR. The latter was derived from electrode Cz

An SNR analysis was performed to analyse the discriminability of CST bandpower with regard to mental tasks. The group-level SNR of CST bandpower as a function of frequency is shown in Fig. 6a. As SNR values are not limited, a robust group-level average was calculated using the median. For visualisation purposes, we smoothed the SNR values before computing the group median with a moving average comprising 5 frequency bands. Due to the bias removal and statistical fluctuations, negative SNR values can occur. One can visually identify CST SNR peaks around 15 and 30 Hz, i.e., in beta and low-gamma bands.

#### Band-averaged and IMC-aligned SNR

We show band-averaged (15–45 Hz) and IMC-aligned CST SNRs in Fig. 6b and Table 3. The band-averaged and IMC-aligned CST SNRs were both statistically significantly larger than their baselines ($W=111$, $p_{adj}=0.003$, and $W=114$, $p_{adj}=0.003$, for band-averaged and IMC-aligned CST SNR, respectively). Moreover, the IMC-aligned CST SNR was statistically significantly larger than the band-averaged CST SNR ($W=12$, $p_{adj}=0.004$). All tests were two-sided Wilcoxon signed-rank tests, and p-values were adjusted for multiple comparisons. The effect of a frequency offset on the IMC-aligned CST SNR is shown in Fig. 6c. The CST SNR peaked around the original IMC peak frequency (offset of 0 Hz), but this peak was not clearly distinct.

#### EEG and CST SNR

Finally, we compared CST and EEG SNR profiles in Fig. 6d. Even though only data from the Laplace filtered EEG channel Cz was used, the EEG SNR was prominent and higher than the CST SNR. This confirms that the employed mental tasks can be detected in macroscale brain oscillations over the motor cortex while an isometric movement is being performed. The EEG SNR peaked in the mu band around 12 Hz and yielded pronounced values up to around 30 Hz. Thus, the motor cortex carries task-informative oscillations, which could leak down to MNs. We chose to use a Laplace filter instead of SPoC because SPoC is a supervised method and depends on the discriminability of the target signal, i.e, the CST. It would result in an ineffective spatial EEG filter at frequencies with a low CST SNR.

**Table 3:** Group-level CST SNR

	Mean (%)	SD (%)	Median (%)
band-averaged	0.02	0.04	0.00
baseline band-averaged	0.00	0.00	0.00
IMC-aligned	0.03	0.05	0.02
baseline IMC-aligned	0.00	0.01	0.00

#### Classification

We extracted features from CST, EEG, and potential confounders from the task phase of trials. The CST and EEG features, *CST-FB* and *EEG-FB*, were bandpower features computed using a filter bank (15–19, 17–21, ..., 41–45 Hz). In addition, we computed CST bandpower features that were aligned with the participants’ IMC peaks (*CST-IMC*). Both *CST-FB* and *CST-IMC* features potentially capture cortical oscillations that leak downstream. The potential confounder features were computed as the mean and standard deviation of the dorsiflexion force and the CST rate (*FORCE-MEAN,* *FORCE-STD* and *CST-RATE*). The four mental tasks were then classified with an sLDA classifier [50, 51] using $10 \times 10$ fold cross-validation.

#### Classification accuracies

**Fig. 7:**
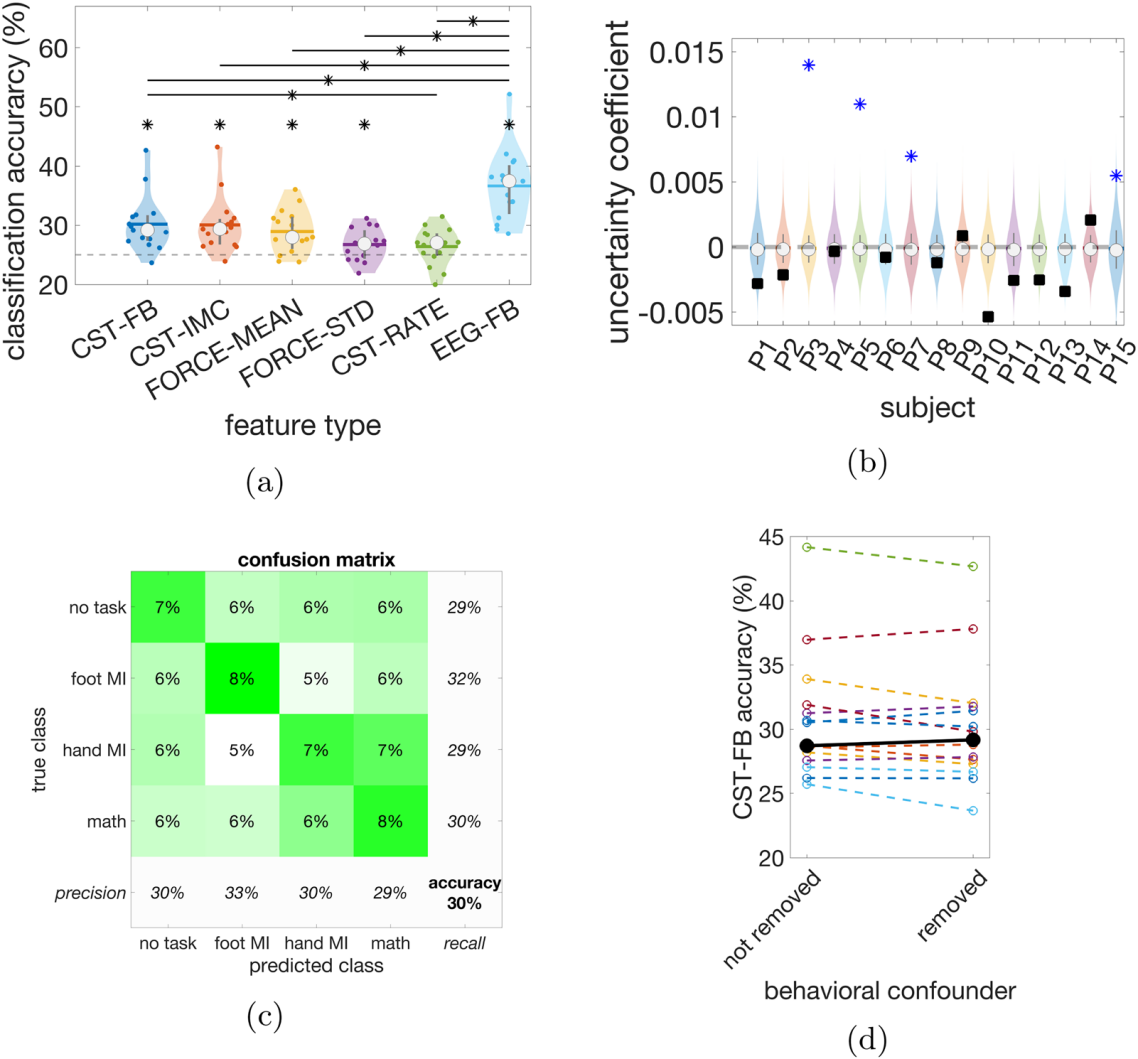
Classification results. **a** Classification accuracies obtained from different features. Each dot represents a participant. The horizontal dashed line indicates the chance level of 25%. The group medians are marked with a circle; the group mean values are marked with a horizontal line; the vertical grey lines indicate the interquartile ranges. A star marks statistically significant differences from the chance level or between groups (two-sided Wilcoxon signed-rank test with $q=0.05$ or Tukey-Kramer post-hoc test with $\alpha =0.05$, respectively). **b** Uncertainty coefficients *U*(*X*, *Y*) between the class predictions from CST-FB and EEG-FB features are shown as black boxes; statistically significant values are indicated with a blue asterisk (one-sided permutation test, $q=0.05$). Additionally, we show the distribution of the uncertainty coefficients when permuting class labels as colored violin plots. **c** Confusion matrix including recall, precision and accuracy values on the sides. The coloured true/predicted fields sum up to 100%. Trials from all participants were pooled. **d** Comparison of CST-FB classification accuracies before and after confounder removal with regression (i.e., classification of residuals). The black dots indicate the medians

The classification accuracies and group statistics are provided in Fig. 7a and Tables 4 and 5. The classification accuracies yielded by CST-FB, CST-IMC, FORCE-MEAN, FORCE-STD and EEG-FB were statistically significantly higher than the chance level of 25% $$(W=118$$, $$p_{adj}={4\text {e}-4}$$; $$W=119$$, $$p_{adj}={4\text {e}-4}$$; $$W=112$$, $$p_{adj}=0.002$$; $$W=96$$, $$p_{adj}=0.050$$; $$W=120,$$ $$p_{adj}={4\text {e}-4})$$. The classification accuracy of CST-rate was not statistically significant $$(W=89$$, $$p_{adj}=0.107)$$. All tests were two-sided Wilcoxon signed-rank tests, and p-values were adjusted for multiple comparisons. The statistical tests show that the oscillations in the CST and EEG signals were modulated by the mental tasks. No strong preference for any particular mental task was present in the confusion matrix for the CST-FB features (Fig. 7c).

The classification accuracies were then compared using a one-way repeated measures ANOVA with feature type as the explanatory variable. A statistically significant difference between classification accuracies was found ($F(5,70)=21.452$, $p={2\text {e}-8}$, GG adjusted). A Tukey-Kramer post-hoc test revealed statistically significant pairwise differences between CST-FB and CST-RATE ($p=0.048$, 95%-CI $=[0.0\%, 7.6\%]$), EEG-FB and CST-FB ($p={2\text {e}-4}$, 95%-CI $=[3.2\%, 9.7\%]$), EEG-FB and CST-IMC ($p={3\text {e}-4}$, 95%-CI $=[3.1\%, 10.1\%]$), EEG-FB and FORCE-MEAN $(p=0.002$, 95%-CI $=[2.8\%, 12.6\%])$, EEG-FB and FORCE-STD ($p={2\text {e}-5}$, 95%-CI $=[5.7\%, 14.1\%]$), and EEG-FB and CST-RATE ($p={1\text {e}-5}$, 95%-CI $=[6.2\%, 14.4\%]$).

The mu-band was excluded from the EEG features because frequencies around 10 Hz are attenuated in spinal circuits [1]. This restricts the classification analysis to cortical oscillations that can potentially propagate to spinal networks, thereby allowing us to focus on CST and EEG signal components that could originate from the same sources. In addition, we employed the same simple feature extraction and classification methods for both CST and EEG signals. While this approach yielded lower EEG classification accuracies than state-of-the-art methods, it enabled a direct comparison between the two signal modalities.

#### Removal of potential confounders

The mental tasks also induced task-dependent changes in force and CST rate features. These task-dependent changes could potentially have confounded the classification analysis of the CST band-power features. Figure 7d shows the CST-FB classification accuracies with and without the removal of these potential confounders using a multiple linear regression (see Methods for details). The removal of these potential confounders did not have a statistically significant effect on the group-level classification accuracy ($W=85$, $p=0.169$, two-sided Wilcoxon signed-rank test). Furthermore, we calculated the median across the pairwise classification accuracy differences (i.e., confounders *not removed* minus *removed*). This yielded a median difference of 0.4% with a 95% confidence interval of CI $=[-0.5\%, 1.1\%]$. The narrow confidence interval around 0% indicates that the removal of potential confounders had a minor impact on the classification accuracies. Thus, it is unlikely that force changes confounded the classification accuracy obtained from CST band-power features.

**Table 4:** Classification accuracies (%) for each participant obtained with different features. Bold values indicate accuracies significantly above chance level (one-sided binomial test [62] accounting for the actual number of trials per participant, with correction for multiple comparisons across all participants and features at a false discovery rate of $q=0.05$ [55])

	P1	P2	P3	P4	P5	P6	P7	P8	P9	P10	P11	P12	P13	P14	P15
CST-FB	**31**	28	**32**	**32**	**43**	24	**38**	26	29	27	28	29	27	30	30
CST-IMC	30	26	**32**	**31**	**43**	24	**37**	26	29	27	29	29	27	30	31
FORCE-MEAN	**34**	**31**	30	**31**	**32**	26	28	25	27	**36**	24	**32**	28	28	24
FORCE-STD	27	25	**30**	27	30	27	**31**	30	26	24	24	24	27	27	22
CST-RATE	27	27	27	29	30	29	**31**	25	25	23	25	22	28	27	20
EEG-FB	**42**	**38**	**38**	**38**	**52**	**35**	**41**	**34**	**37**	30	29	**34**	**31**	**41**	29

**Table 5:** Group statistic of the classification accuracies obtained with different features

	Mean (%)	SD (%)	Median (%)
CST-FB	30	5	29
CST-IMC	30	5	29
FORCE-MEAN	29	4	28
FORCE-STD	27	3	27
CST-RATE	26	3	27
EEG-FB	37	6	37

#### CST vs. Force and EEG

**Fig. 8:**
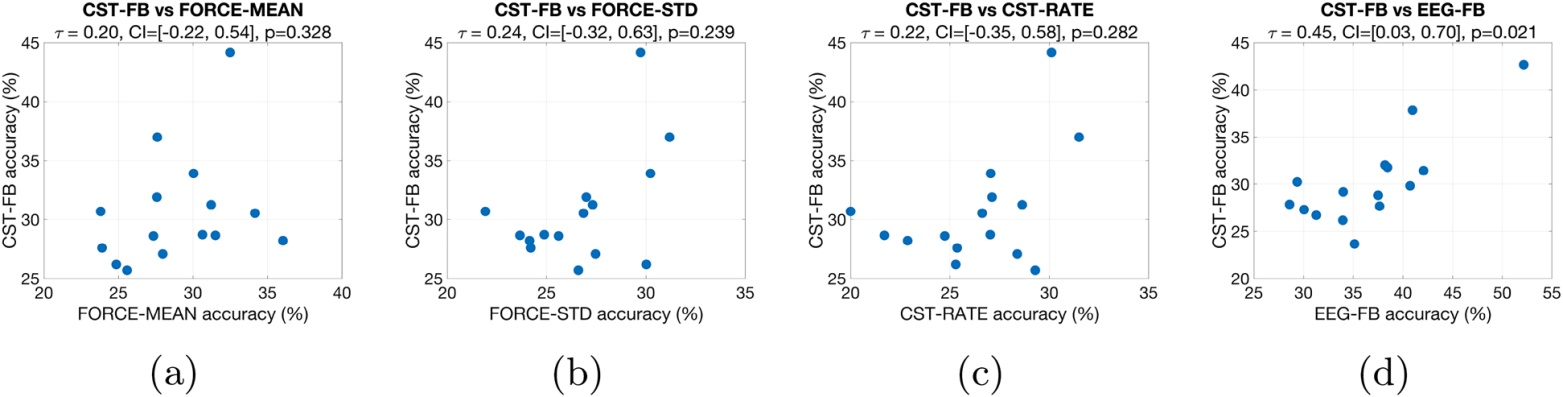
Correlation plot of classification accuracies. The Kendall rank correlation coefficient $\tau$ of the classification accuracies and its 95% confidence interval and p-value are shown in the title. **a-c** Comparisons of features capturing force output **d** Comparison with EEG (note the altered scale)

Mental tasks could be classified from force-related features (force mean value, force standard deviation, and CST rate). We investigated whether there was a correlation between the classification accuracies yielded by CST band-power features and force-related features (Fig. 8a–c). Importantly, the effect of force and CST rate on the CST-FB features was not removed here to reveal any potential correlation of the raw features. We provide the Kendall rank correlation coefficients $\tau$ of the classification accuracies, associated p-values (two-sided permutation test), and 95% confidence intervals in the plots. The results did not indicate any apparent correlation of classification accuracies between CST-FB features and the three force-related features. In line with that, the Kendall rank correlation coefficients were not statistically significant. Note, however, that confidence intervals were large, and, thus, the available data could not demonstrate the absence of correlation. Between CST and EEG band-power features, a statistically significant correlation of $\tau =0.45$ ($p=0.021$) was observed (Fig. 8d CST-FB vs. EEG-FB).

#### Predicted class labels from CST and EEG

We further analysed the association between the predicted class labels from CST-FB and EEG-FB features with the uncertainty coefficient *U*(*X*, *Y*) [52] (see Fig. 7b). Statistically significant coefficients were obtained for participants P3 ($p_{adj}={1\text {e}-4}$), P5 ($p_{adj}={1\text {e}-4}$), P7 ($p_{adj}={1\text {e}-4}$), and P15 ($p_{adj}=0.034$) (one-sided permutation tests adjusted for multiple comparisons across participants at $q=0.05$). Thus, for these participants, the brain signals and the CST yielded related class predictions for individual trials.

### Force output

#### Task-dependent differences in force-related parameters

**Fig. 9:**
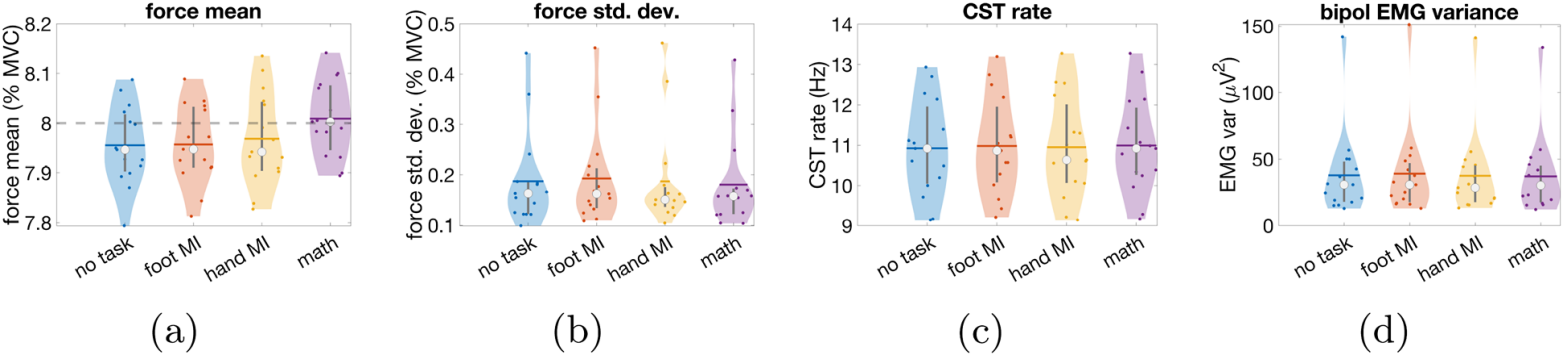
Force-related parameters during the task phase (6–9.5 s after trial start) as a function of the mental task. The data points are the participants’ medians over trials for the respective parameter and task. The group medians are marked with a circle; the group mean values are marked with a horizontal line; the vertical grey lines indicate the interquartile ranges. **a** Mean value of the force. The horizontal dashed line marks the target force at 8%. **b** Standard deviation of the force. **c** CST rate. **d** EMG variance of an antagonist muscle (medial head of the right gastrocnemius muscle)

We analysed four parameters reflecting force changes, i.e., force mean, force std. dev., CST rate and bipolar EMG variance. The bipolar EMG variance was derived from an antagonist muscle (medial head of the right gastrocnemius muscle) after 10 Hz high-pass filtering. The parameter values are shown in Fig. 9 for the investigated mental tasks. Figure 9a suggests that participants applied a marginally greater force during the math task compared to the other tasks. The group average for *math* was up to 0.05% higher than for the other tasks. Interestingly, the force in the other tasks *no task*, *foot MI*, and *hand MI* was, on average, between 0.03% and 0.04% below the target force. In contrast, the group average in the math task was 0.01% above the target force. No apparent differences were observed for the other parameters (Fig.  9b–d).

The standard deviation of the force was high for three participants in each task (see Fig. 9b). For *no task*, *hand MI*, and *math*, these participants were consistently P6, P15, and P11, listed in order of decreasing standard deviation. In the case of *foot MI*, P6, P15 and P7 had the highest standard deviation. P6, P15, and P11 did not consistently achieve a high classification accuracy for CST-FB or FORCE-STD features (c.f. Table 4). Only P7 achieved a notably high classification accuracy for CST-FB at 38% (31% for FORCE-STD).

We compared the force-related parameters shown in Fig. 9 across the mental tasks. Each parameter was treated as a separate response variable, and we conducted four separate one-way repeated measures ANOVAs with mental task as the explanatory variable. The p-values were adjusted for multiple comparisons. A statistically significant difference between mental tasks was found for force mean $(F(3,42)=12.379$, $p_{adj}={2\text {e}-5}),$ whereas no significant differences were found for force std. dev. ($F(3,42)=1.565$, $p_{adj}=0.283$), CST rate ($F(3,42)=0.959$, $p_{adj}=0.421$), or bipolar EMG variance ($F(3,42)=2.882$, $p_{adj}=0.182$, GG adjusted).

#### Independence of task effects on CST bandpower from force

The observed task-dependent force mean changes could have caused the observed CST bandpower modulations. We therefore applied the regression approach described in section “Independence of task effects on CST bandpower from force” to test whether the task effect on CST bandpower remained after controlling for force. Task had a significant effect on CST bandpower after controlling for force ($\Delta R^2_{adj}$: median $= 0.013$, 95%-CI $=[0.007, 0.023]$, $W=116$, $p_{adj}={6\text {e}-04}$). Furthermore, task and CST bandpower were significantly associated without conditioning on force (marginal adjusted $R^2$: median $= 0.013$, 95%-CI $=[0.007, 0.024]$, $W=115$, $p_{adj}={6\text {e}-04}$), ruling out that the observed task effect arose from conditioning on a collider. Two-sided Wilcoxon signed-rank tests corrected for multiple comparisons [55] were used. These results show that task had an effect on CST bandpower and this effect was not fully mediated by force. These analyses assumed linear relationships between variables and additivity.

#### EEG ERD/S plots

**Fig. 10:**
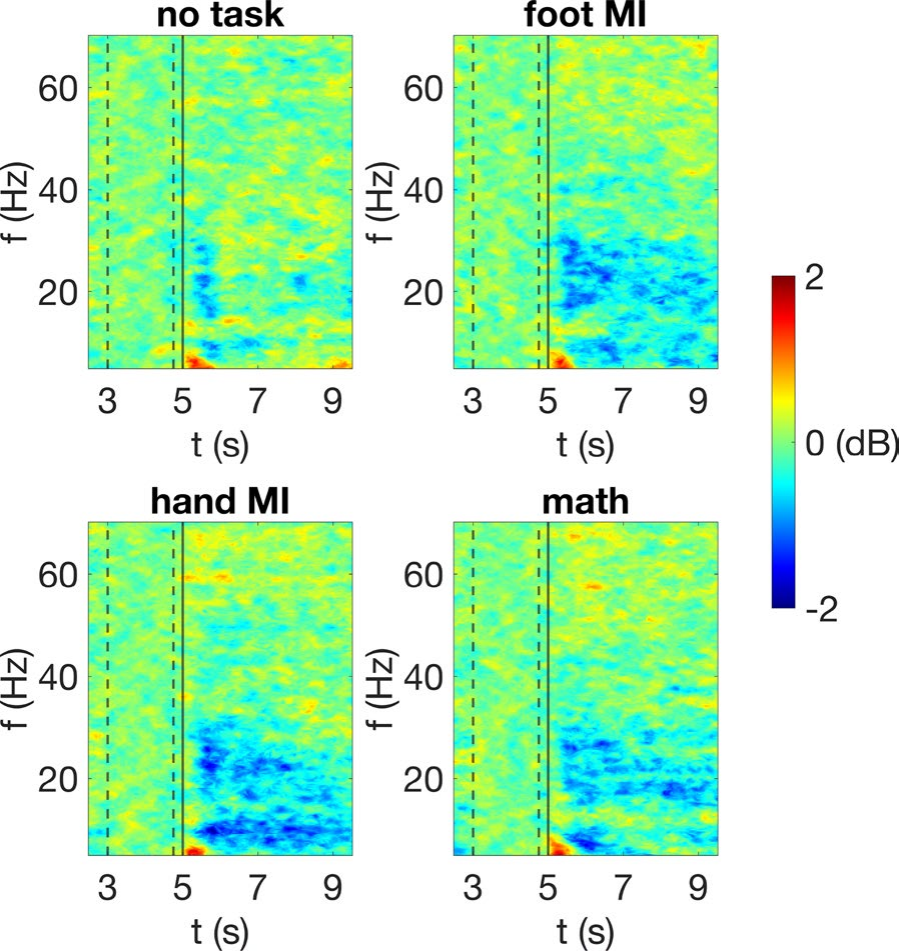
Group-level ERD/S maps of the four mental tasks at electrode Cz. The task cue was at second 5. Colors depict power relative to the baseline interval ranging from 3 s to 5 s as indicated by the dashed lines

The event-related desynchronization/synchronisation (ERD/S) plots for the four mental tasks are shown in Fig. 10. We computed the ERD/S using an STFT with a 1-second Hanning window and the gain model [33, 63]. The plots depict the median across all participants of the Laplace-filtered central EEG electrode Cz. The baseline interval ranged from 3 to 5 s and included the isometric dorsiflexion of the right foot. This minimised the impact of the executed isometric movement on the ERD/S plots.

A relative power decrease, i.e., ERD, occurred shortly after cue presentation in the mu and beta bands for all four tasks. In the *no task* condition, there was no pronounced ERD or ERS observable after 6 s. In contrast, *foot MI*, *hand MI*, and *math* exhibited an ERD in the beta band between 15 Hz to 30 Hz. Additionally, there was an ERD in the mu band around 10 Hz for *foot MI* and particularly *hand MI*. The ERD shortly after cue presentation could reflect the processing of the external stimulus. The pronounced ERD in *foot MI*, *hand MI*, and *math* after 6 s is likely related to the execution of the respective mental task.

## Discussion

We found that *power modulations* of cortical beta and low-gamma oscillations induced by mental tasks were accompanied by concordant power modulations in the cumulative spike train of spinal motor neurons. The mental tasks were distinct from the executed movement required to maintain CST activity (sustained dorsiflexion). Furthermore, the task effect on CST bandpower was not fully accounted for by force, and the observed EEG-CST bandpower correlations were not explained by potential force-related confounders. The identified EEG sources mostly aligned with the foot area of the primary motor or somatosensory cortex. These findings support the hypothesis that cortical oscillations above 10 Hz that do not contribute to ongoing motor control can propagate downstream to MNs and are not removed by spinal networks.

### Connectivity analyses

The transmission of beta and low-gamma oscillations from the motor cortex to MNs is well-documented with connectivity analyses such as corticomuscular coherence [8–14], directed coherence [64] or directed transfer function [65]. Furthermore, Bräcklein et al. discovered that beta bursts at the cortical and MN levels have the same source, suggesting that MN beta oscillations result from cortical projections [66], such as corticomotoneuronal connections [18–20]. It has also been found that the simultaneous execution of additional motor or non-motor tasks reduces CMC [67–69]. However, in contrast to previous studies, we were not investigating EEG-CST connectivity per se, but whether brain and MN oscillations comodulate in power across mental tasks due to the existing connectivity. In contrast to our findings, [67–69] did not observe trial-related (spontaneous) or task-related band-power comodulations between EMG and EEG signals. This could be because EMG signals are susceptible to interference between motor unit action potentials, potentially masking bandpower changes, whereas the CST is not. Moreover, we employed a supervised data-driven spatial EEG filter, which typically offers better SNR compared to a fixed spatial filter. Furthermore, while [67–69] recorded EMG from hand muscles, we derived MN activities from the TA muscle. It remains to be seen in future studies whether the observed power comodulation generalises to other muscles, including hand muscles.

### Spatial and frequency characteristics of transmitted oscillations

CST bandpower was particularly informative about the mental task around the IMC peak frequency, and bandpower correlations between CST and EEG were also largest at this frequency. The IMC reflects the strength of the common synaptic input, and the common synaptic input is at least partly of supraspinal origin [7, 10, 70–72]. Thus, the strongest EEG-CST bandpower correlation occurred at a frequency where the common synaptic input, and so the cortical input, is believed to be high. Furthermore, the SPoC patterns suggest a centrally located dipole as a source, potentially in the foot area of the primary motor or somatosensory cortex. This finding is consistent with the somatotopic arrangement of CMC [8, 73–75]. Both findings indicate that CST oscillations modulated by mental tasks originated from the cortex.

The EEG SNR profile did not resemble the CST SNR profile. The EEG SNR was highest around the mu band, whereas the CST had low SNR in the mu band. This is not surprising as the cortical mu band is particularly modulated by motor imagery tasks [21], but supraspinal signals in the mu band are suppressed at the MN level [3, 4].

For the mental tasks we studied, the discriminability of the EEG signal extended over the mu, beta, and low-gamma bands. However, the motor cortex oscillations modulated by these mental tasks were not transmitted downstream in a broadband manner, but preferentially around the IMC peak frequency.

### Using CST bandpower as a control signal for neural interfacing applications

Mental tasks could be decoded from CST features significantly above chance. However, the average classification accuracy was 30% (chance level 25%). A substantial improvement in classification performance is necessary to derive a viable control signal from task-dependent CST oscillations. We employed a shrinkage LDA classifier, which is well-suited for comparable BCI experiments with oscillatory cortical signals obtained using EEG [76]. Other classifiers, such as support vector machines or artificial neural networks, may improve performance, but the performance gain is expected to be moderate [76, 77]. A more promising approach is intensive user training, which has been shown to substantially improve BCI performance [78–81]. Initially, users would employ mental tasks as a control strategy to modulate CST bandpower. As they receive feedback on CST bandpower, they would learn to control it more accurately over the course of several weeks. In [44], neurofeedback-based training using CST beta bandpower has been tested for a single session. Participants received visual feedback of their beta bandpower but were not told any explicit mental strategy on how to alter bandpower. Combining such feedback training with an initial mental strategy based on explicit mental tasks could help acquire control skills more quickly.

Our investigation focused on CST signals obtained from the TA muscle, but CSTs from other muscles likely contain task-related information as well. Multi-muscle signal fusion could therefore represent another avenue for enhancing classification accuracy.

If classification performance can be substantially improved, mental-task-based control using CST signals decoded in quasi-real-time [82] could provide simple commands for movement augmentation with artificial limbs [31] or for motor neuroprostheses in individuals with spinal cord injury [29]. For the latter application, even clinically complete injuries can preserve some MN activity below the lesion level, and mental-task-based control could provide an alternative or complementary control signal to MN firing rates [30]. Additionally, combining HD-EMG with other neural signals, such as EEG, may yield more robust bandpower-based control signals when using mental-task paradigms. Future research should determine whether CST signals provide complementary or redundant information compared to non-invasively recorded brain signals.

However, there is a fundamental limitation: muscle activation is required to generate the MN activity from which CST bandpower is derived. Future work could explore minimising this constraint through muscle coactivation strategies that provide the necessary background MN activity while maintaining postural stability [83].

### Task-dependent force output

Beta oscillations do not directly translate into changes in muscle force [7]. However, the reverse is in general not true, and task-dependent force output could have given rise to the observed modulations in beta oscillations. Additionally, dual-task interference [84], for example, through competition for shared mental resources, could have induced common (dependent) bandpower changes in CST and EEG.

We found significant differences between mental tasks in force at the group level, and it was possible to classify tasks based on force features. However, a regression analysis showed that the task effect on CST bandpower was not fully mediated by force. This rules out that the observed task-discriminative CST bandpower modulations were solely a consequence of task-dependent force changes. This conclusion relies on the linearity and additivity assumption of the regression model. We also removed the effect of force and CST rate on CST bandpower using multiple linear regression, retaining only the residuals for the analyses. Since force-related variance was removed from CST bandpower before computing the EEG–CST correlations, the significant correlations between the residual CST bandpower and EEG ($\tau _{trials}$ and $\tau _{tasks}$) demonstrate that EEG and CST bandpower shared variance beyond what can be attributed to force. This holds as long as the relationship between CST bandpower and force is linear. Even if this assumption is not fully met, the conclusion is further supported by the observation that the EEG–CST bandpower correlations were larger than the correlations between EEG and force-related parameters and that correlations between EEG and force-related parameters were close to zero. Note that this approach based on residuals eliminated only the effect of measurable force changes. Potential force changes along axes not captured by the force sensor remained unaccounted for. Additionally, force changes might have been masked by the unavoidable slack in the strap securing the foot to the force sensor. However, removing the effects of force and CST rate had no impact on classification accuracy, indicating that these variables did not significantly confound CST bandpower in the first place. The classification accuracies obtained from force features and CST bandpower features were furthermore uncorrelated across participants, providing additional evidence that task-dependent force changes do not explain the classification results obtained with CST bandpower features.

Moreover, CST and EEG beta power were modulated in the same direction across tasks (positive correlation). Task-dependent force changes are unlikely to account for this pattern, as EEG beta power is not sensitive to force during movement [85–87]. Consistent with this, the task correlations between EEG and force-related parameters were close to zero at the group level, even when using spatial filters optimised to extract these force-related parameters.

The observed correlations between CST and EEG were not solely driven by bandpower differences between the single-task condition (*no task*) and the dual-task conditions (*foot MI*, *hand MI*, or *math* task), as excluding *no task* still yielded significant CST-EEG correlations. Possible differences in task difficulty between the dual-task conditions are also unlikely to explain the correlations, as EEG beta and gamma bands are not sensitive to task difficulty in dual-task paradigms [88].

In contrast to force, the CST rate was not predictive of the mental task. This is surprising because the CST rate reflects the neural drive to the muscle and its force output. One possible reason is that MN decompositions over-represent MNs with higher recruitment thresholds, leading to an inaccurate estimation of the neural drive to the muscle [89]. In addition, at low forces, as in this experiment, changes in force are mainly driven by changes in MN recruitment rather than changes in MN rates [90]. The CST rate may have remained largely unaltered by recruitment changes, as we only considered a fixed set of predominantly active MNs when calculating the CST rate.

In conclusion, although force mean differed across mental tasks, the task-dependent CST bandpower modulations and their correlation with EEG signals were not explained by force changes.

### Limitations

It is unclear whether beta-band coherence [8–14] arises as a consequence of the downstream propagation of oscillations not contributing to ongoing force control, or whether it reflects a carrier mechanism that is a prerequisite for their propagation. If the latter is true, the decreased coherence during dynamic movements [12, 75, 91, 92] could impair downstream propagation of these oscillations. This would limit the applicability of CST bandpower as a control signal for neural interface applications such as movement augmentation.

The information flow between the cortex and MNs in the beta band was found to be bidirectional by Witham et al. [64]. Our analysis approach based on power correlation is unable to determine the directionality of information flow. While it is possible that some oscillations might be transmitted back to the cortex, this particular question is beyond the scope of this paper. Furthermore, our methodology cannot exclude that the found correlations between EEG and CST result from a common input from a third, possibly subcortical source.

Recent research suggests that sensorimotor beta, appearing as sustained oscillation in trial averages, may actually consist of discrete beta bursts at the single-trial level [93, 94]. Beta bursts at the cortical and MN levels were found to be time-locked [66, 95] and were similarly altered in a neurofeedback scenario [66]. Our analyses are based on the variance of the EEG and CST within the task phase. Variance reflects the power within this period and is monotonically related to the amplitude of sustained oscillations or, for bursts, to their rate, duration, or amplitude. Given this monotonic relationship, the identified comodulation between CST and EEG is consistent with downstream propagation of cortical oscillations independent of force output, regardless of whether they manifest as sustained oscillations or bursts. However, in the case of bursts, we cannot exclude the possibility that different burst parameters were comodulated. For example, CST burst *duration* may have been comodulated with EEG burst *amplitude*. Such potential cross-parameter comodulation would imply an intermediate processing step, possibly implemented in spinal networks, although its functional role would be unclear.

## Conclusion

The execution of mental tasks induced common power changes in macroscale brain signals and spinal motor neurons. Furthermore, these power changes were discriminable across mental tasks at the level of spinal motor neurons. As the mental tasks were distinct from the executed motor task, our findings are consistent with the notion that cortical oscillations not contributing to ongoing force control can propagate to spinal motor neurons.

## Data Availability

Source data for this study are not publicly available due to privacy restrictions.

## Footnotes

1 https://yaga.readthedocs.io

2 https://github.com/sccn/labstreaminglayer

3 https://github.com/neurofreiburg/QuattrocentoLSLApp

## Declarations

### AI usage

Claude (Anthropic) and ChatGPT (OpenAI) were used to assist with statistical reasoning, interpretation, and coding. All outputs were critically reviewed and verified and all statistical analyses were performed by the authors, who take full responsibility for the analyses and conclusions. The authors also used Claude and ChatGPT to improve the language and readability of the text. The manuscript was written by the authors, who reviewed and approved all revisions and take full responsibility for the final published version.
